# Identification of novel tylosin analogues generated by a *wblA* disruption mutant of *Streptomyces ansochromogenes*

**DOI:** 10.1186/s12934-015-0359-5

**Published:** 2015-11-02

**Authors:** Cheng Lu, Guojian Liao, Jihui Zhang, Huarong Tan

**Affiliations:** State Key Laboratory of Microbial Resources, Institute of Microbiology, Chinese Academy of Sciences, Beijing, 100101 China; University of Chinese Academy of Sciences, Beijing, 100049 China; College of Pharmaceutical Sciences, Southwest University, Chongqing, 400715 China

**Keywords:** *wblA*, Nikkomycin, Tylosin analogues, *Streptomyces ansochromogenes*, Bioassay

## Abstract

**Background:**

*Streptomyces*, as the main source of antibiotics, has been intensively exploited for discovering new drug candidates to combat the evolving pathogens. Disruption of *wblA*, an actinobacteria-specific gene controlling major developmental transition, can cause the alteration of phenotype and morphology in many species of *Streptomyces*. One *wblA* homologue was found in *Streptomyces ansochromogenes* 7100 by using the Basic Local Alignment Search Tool. It is interesting to identify whether novel secondary metabolites could be produced by the *wblA* disruption mutant as evidenced in other *Streptomyces*.

**Results:**

The *wblA* disruption mutant of *S. ansochromogenes* 7100 (ΔwblA) was constructed by homologous recombination. ΔwblA failed to produce spores and nikkomycin, the major product of *S. ansochromogenes* 7100 (wild-type strain) during fermentation. Antibacterial activity against *Staphylococcus aureus* and *Bacillus cereus* was observed with fermentation broth of ΔwblA but not with that of the wild-type strain. To identify the antibacterial compounds, the two compounds (compound **1** and compound **2**) produced by ΔwblA were characterized as 16-membered macrolides by mass spectrometry and nuclear magnetic resonance spectroscopy. The chemical structure of these compounds shows similarity with tylosin, and the bioassays indicated that the two compounds inhibited the growth of a number of gram-positive bacteria. It is intriguing that they displayed much higher activity than tylosin against *Streptococcus pneumoniae*.

**Conclusions:**

Two novel tylosin analogues (compound **1** and **2**) were generated by ΔwblA. Bioassays showed that compound **1** and **2** displayed much higher activity than tylosin against *Streptococcus pneumoniae*, implying that these two compounds might be used to widen the application of tylosin.

**Electronic supplementary material:**

The online version of this article (doi:10.1186/s12934-015-0359-5) contains supplementary material, which is available to authorized users.

## Background

The crisis of antibiotic resistance has become an impending global problem, so novel antibiotics are required to combat the evolving pathogens and new emerging diseases. More than half of medically important antimicrobial and antitumor antibiotics are produced by *Streptomyces*. Genome engineering and gene manipulation on secondary metabolic gene clusters have been widely applied for exploring novel bioactive agents. For example, using heterologous expression, a 157 kb daptomycin biosynthetic gene cluster from *Streptomyces roseosporus* NRRL 15998 was successfully cloned and heterologously expressed in *Streptomyces coelicolor* [1]. Two hybrid antibiotics were generated by genetic manipulation of the nikkomycin and polyoxin biosynthetic gene clusters [2]. Supplementation of the mutasynthesis strain with nicotinic acid led to the production of two novel nikkomycin analogues [3]. However, sequencing of several *Streptomyces* genomes revealed that a large number of antibiotic biosynthetic gene clusters are present, which have the potential to produce many more natural products than had previously been recognized [4–7]. Therefore, it has become necessary to devise methods and strategies to identify valuable natural products. One of the features of antibiotic synthesis in *Streptomyces* is that the production of antibiotics is generally associated with the development and differentiation of *Streptomyces*. Genetic manipulations of pleiotropic regulators responsible for both differentiation and antibiotic production may effectively influence the expression of certain genes involved in metabolic pathways, thus it would be an efficient strategy for searching novel metabolites. By this approach, comprehensive elucidations on biosynthetic pathways or regulatory mechanisms of the metabolite biosynthesis could be circumvented.

*whi* genes are involved in the life cycle of *Streptomyces* as well as in the production of various antibiotics [8]. Disruption of these genes resulted in white phenotype of aerial hyphae in *Streptomyces*, so they were named as *whi* genes. *whiB* gene was originally discovered in *Streptomyces coelicolor*, and *whiB*-like (*wbl*) genes are widespread in *Streptomyces* [9, 10]. There are at least 11 homologues of *whiB* genes on the chromosome of *S. coelicolor*. Mutation or absence of *wblA* caused multiple effects on *Streptomyces*, such as the failure of sporulation, enhancement of actinorhodin, undecylprodigiosin, doxorubicin, tautomycetin, and moenomycin production [9, 11–13]. Therefore, WblA is recognized as a global regulator. It plays as a repressor of antibiotic production in *S. coelicolor*, but acts as a pivotal activator for natamycin biosynthesis in *Streptomyces chattanoogensis* L10 [14]. *Streptomyces ansochromogenes* 7100, a natural peptidyl nucleoside antibiotic nikkomycin producer, has been studied for decades [15]. Like other well-studied *Streptomyces*, it has a typical life cycle of differentiation and development with aerial mycelia and spore formation accompanied by secondary metabolites biosynthesis. In search of the sequenced genome of *S. ansochromogenes*, a *whiB*-like gene situated on the chromosome was found and its encoding protein shares 96 % identity with WblA in *S. coelicolor*, likewise it was named as *wblA* (gene accession number KT583835).

In this study, we focused on the secondary metabolites produced by the *wblA* disruption mutant of *S. ansochromogenes* 7100 (ΔwblA). It is intriguing that ΔwblA failed to produce nikkomycin but led to the discovery of novel active metabolites simultaneously. These compounds were subsequently isolated, purified and analyzed for their structures and bioactivities against a number of bacteria.

## Results

### Construction of *wblA* disruption mutant and its complementation

**Fig. 1:**
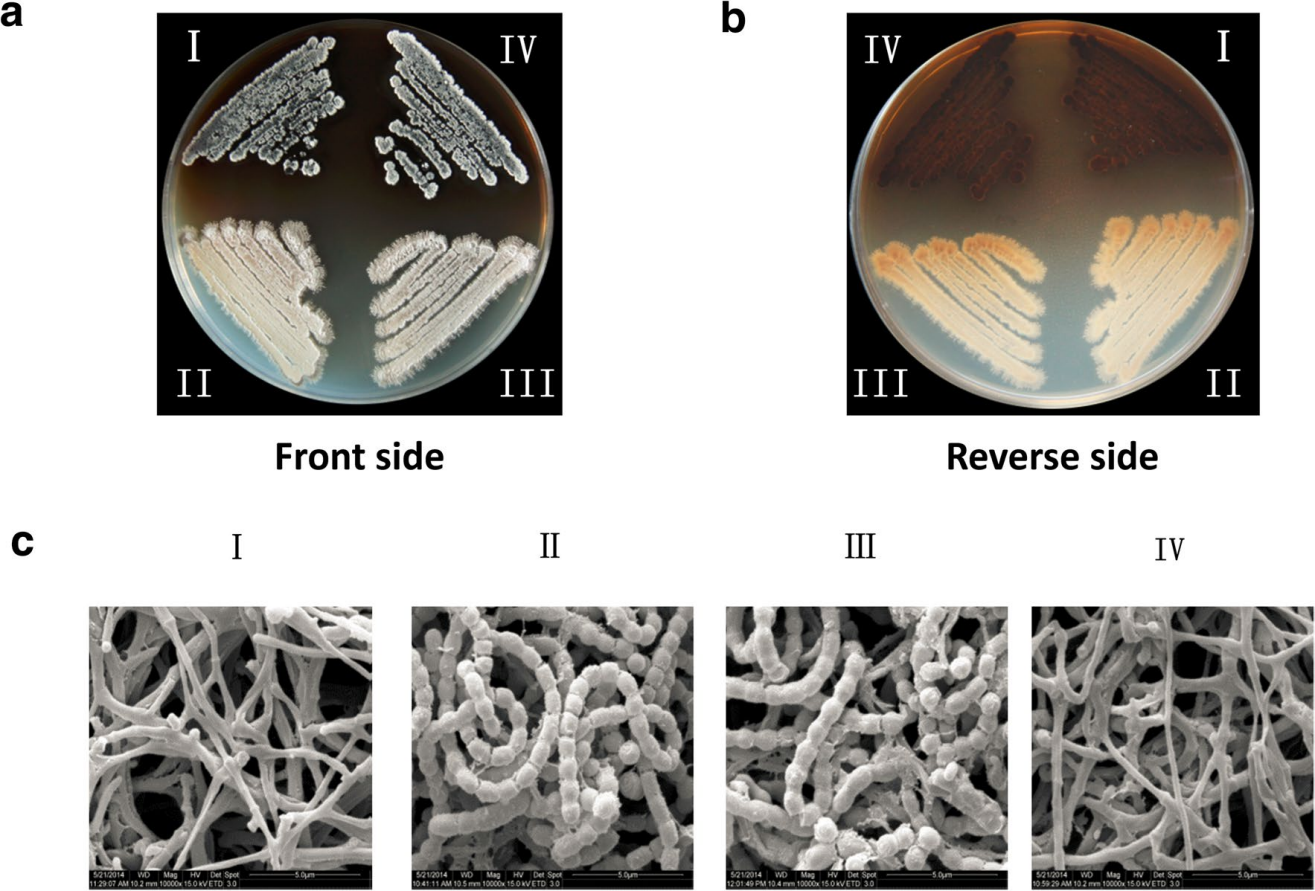
Effects of *wblA* disruption on the phenotype and morphological differentiation of *S*. *ansochromogenes* 7100. Observations on the phenotype of *S. ansochromogenes* 7100 and its derivatives from both sides of the plate (**a**, **b**), and the scanning electron micrographs of the mycelia and spores (**c**). (*I*): ΔwblA, (*II*): *S*. *ansochromogenes* 7100, (*III*): complemented strain by integrating a copy of *wblA* into the chromosome of ΔwblA, (*IV*): the control strain by integrating pSET152 vector into the chromosome of ΔwblA

**Fig. 2:**
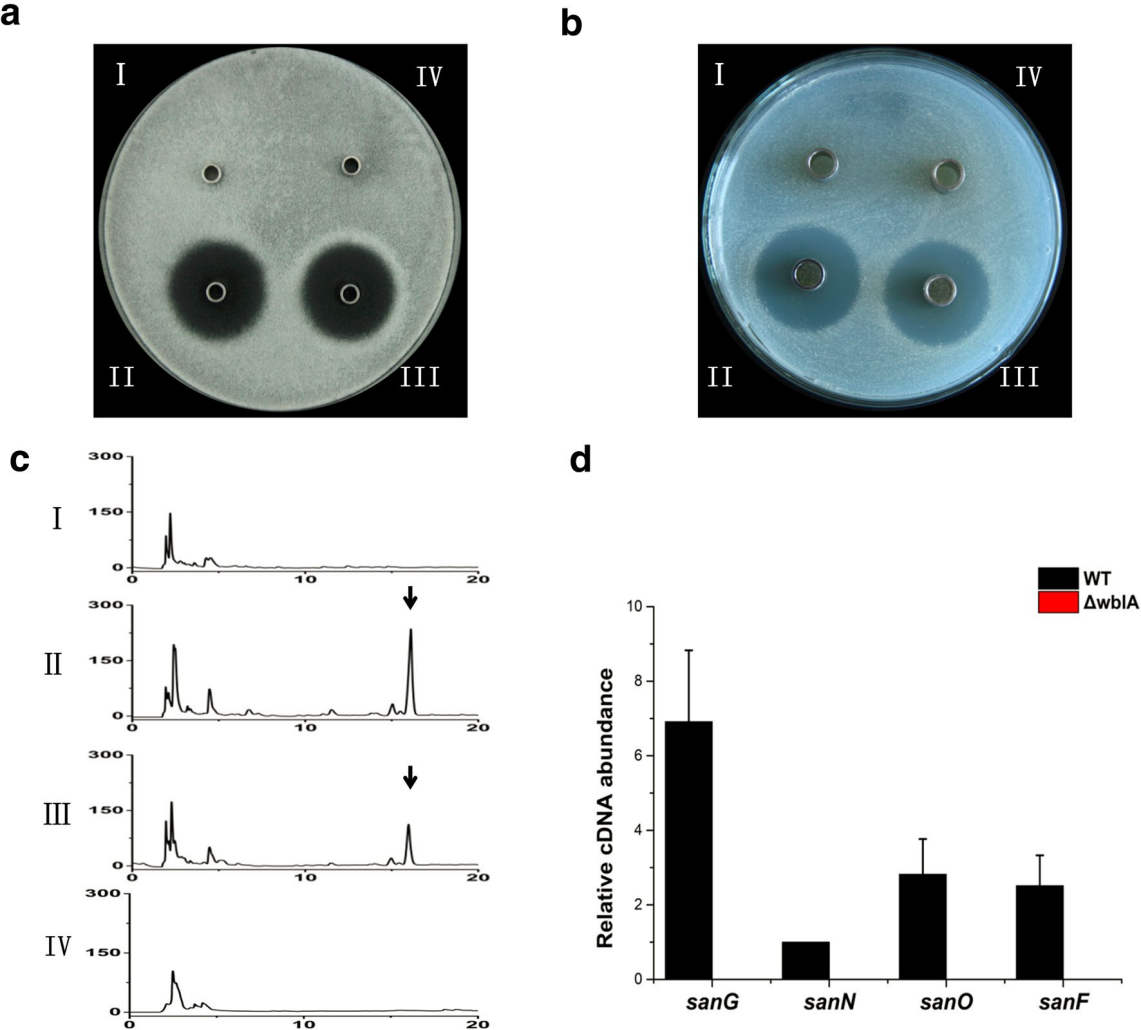
Effects of *wblA* disruption on nikkomycin production. **a** The bioassay of nikkomycin against *Alternaria longipes*. **b** The bioassay of nikkomycin against *Candida albicans*. **c** HPLC analysis of nikkomycin. **d** Transcription analysis of genes related to nikkomycin biosynthesis by qRT-PCR; the transcript of *hrdB* was used as an internal control. (*I*): ΔwblA, (*II*): *S*. *ansochromogenes* 7100, (*III*): complemented strain by integrating a copy of *wblA* into the chromosome of ΔwblA, (*IV*): the control strain by integrating pSET152 vector into the chromosome of ΔwblA. *Arrows* indicate the peak of nikkomycin on HPLC produced by *S. ansochromogenes* 7100

In order to identify whether the metabolite profile could be affected by *wblA* disruption in *S. ansochromogenes* 7100, ΔwblA was constructed via homologous recombination. As expected, ΔwblA failed to form grey spores and spore chains on minimal medium (MM) agar in comparison with wild-type (WT) strain (Fig. 1a–c). On the other hand, nikkomycin, the only secondary metabolite identified so far from this strain, was examined. Cultures from the same time-course experiments were subjected to bioassays against *Alternaria longipes* and *Candida albicans* for nikkomycin activity test (Fig. 2a, b). In contrast to WT strain, no inhibition zone was observed against above two indicator strains with the fermentation filtrate of ΔwblA. High-performance liquid chromatography (HPLC) analysis demonstrated that the production of nikkomycin was completely abolished in ΔwblA (Fig. 2c). To further verify the effect of *wblA* disruption on nikkomycin production, the transcription profile of genes involved in nikkomycin biosynthesis was analyzed by quantitative Real Time Polymerase Chain Reaction (qRT-PCR). The biosynthetic gene cluster of nikkomycin includes one pathway-specific regulatory gene (*sanG*) and 21 structural genes consisting of three transcriptional units (*sanO*-*V, sanN*-*I and sanF*-*X*) [16]. The first gene of each transcriptional unit was chosen to examine the transcription of corresponding genes. The results showed that transcriptions of *sanG* and other three genes (*sanN*, *sanO* and *sanF*) situated in each transcriptional unit were all not detected in ΔwblA, whereas the transcription of *hrdB* as internal control, encoding the principal sigma-like factor, was not affected by the disruption of *wblA* (Fig. 2d). Complementary experiment was performed by integrating a copy of *wblA* and pSET152 vector into the chromosome of ΔwblA, respectively. As expected, nikkomycin production in ΔwblA was restored as that in WT strain (Fig. 2a–c). These results demonstrated that *wblA* is essential for nikkomycin biosynthesis in *S. ansochromogenes* 7100. Disruption of this gene affected not only the spore formation but also the nikkomycin biosynthesis, implying that *wblA* possesses multiple functions.

### Analyses of the secondary metabolites of ΔwblA

**Fig. 3:**
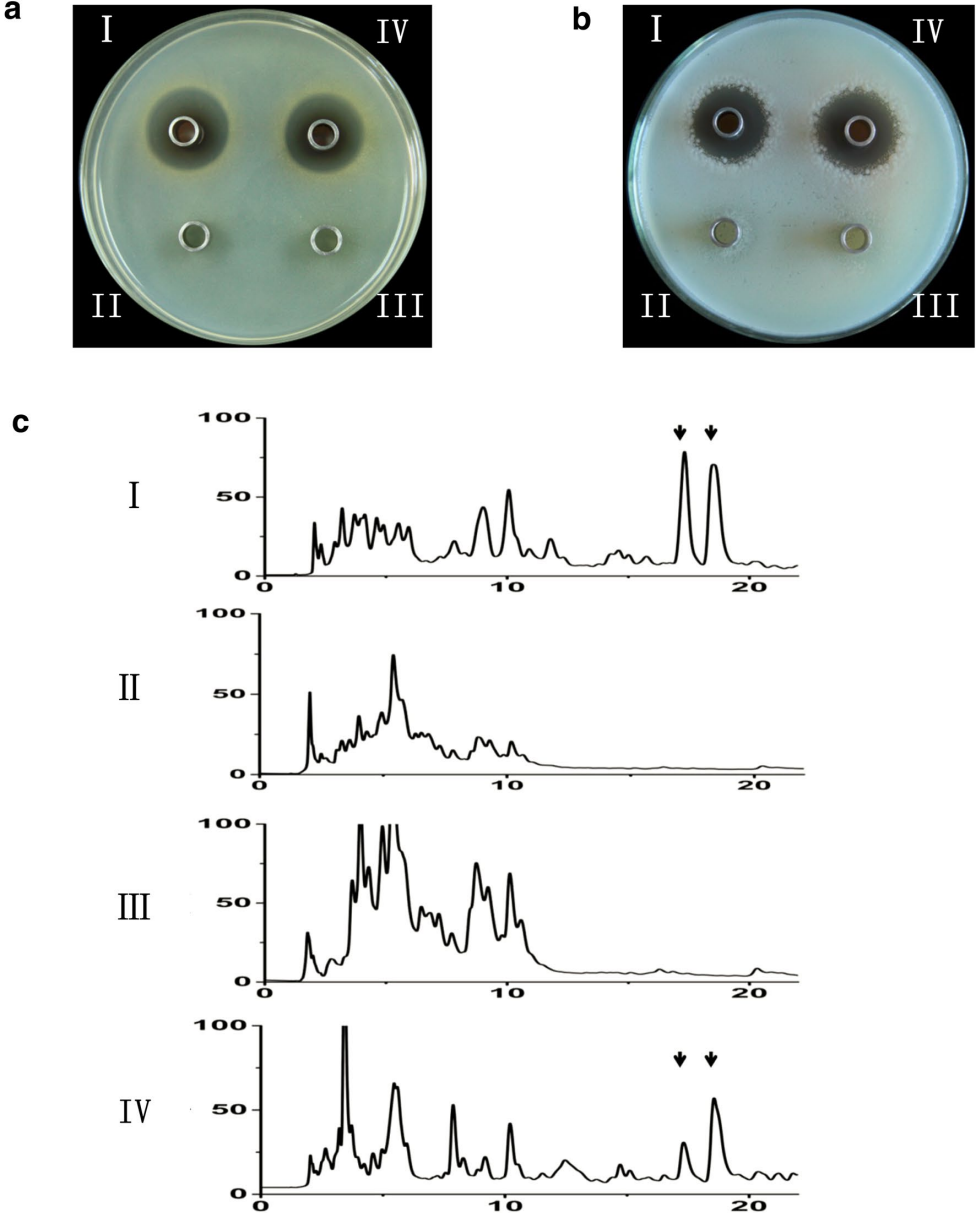
Bioassays and HPLC analysis of the fermentation broth from *S*. *ansochromogenes* 7100 and ΔwblA. Bioassays of the fermentation broth against *Staphylococcus aureus* (**a**) and *Bacillus cereus* (**b**), and the HPLC analysis (**c**). (*I*): ΔwblA, (*II*): *S*. *ansochromogenes* 7100, (*III*): complemented strain by integrating a copy of *wblA* into the chromosome of ΔwblA, (*IV*): the control strain by integrating pSET152 vector into the chromosome of ΔwblA. *Arrows* indicate the new appeared peaks on HPLC produced by ΔwblA

**Fig. 4:**
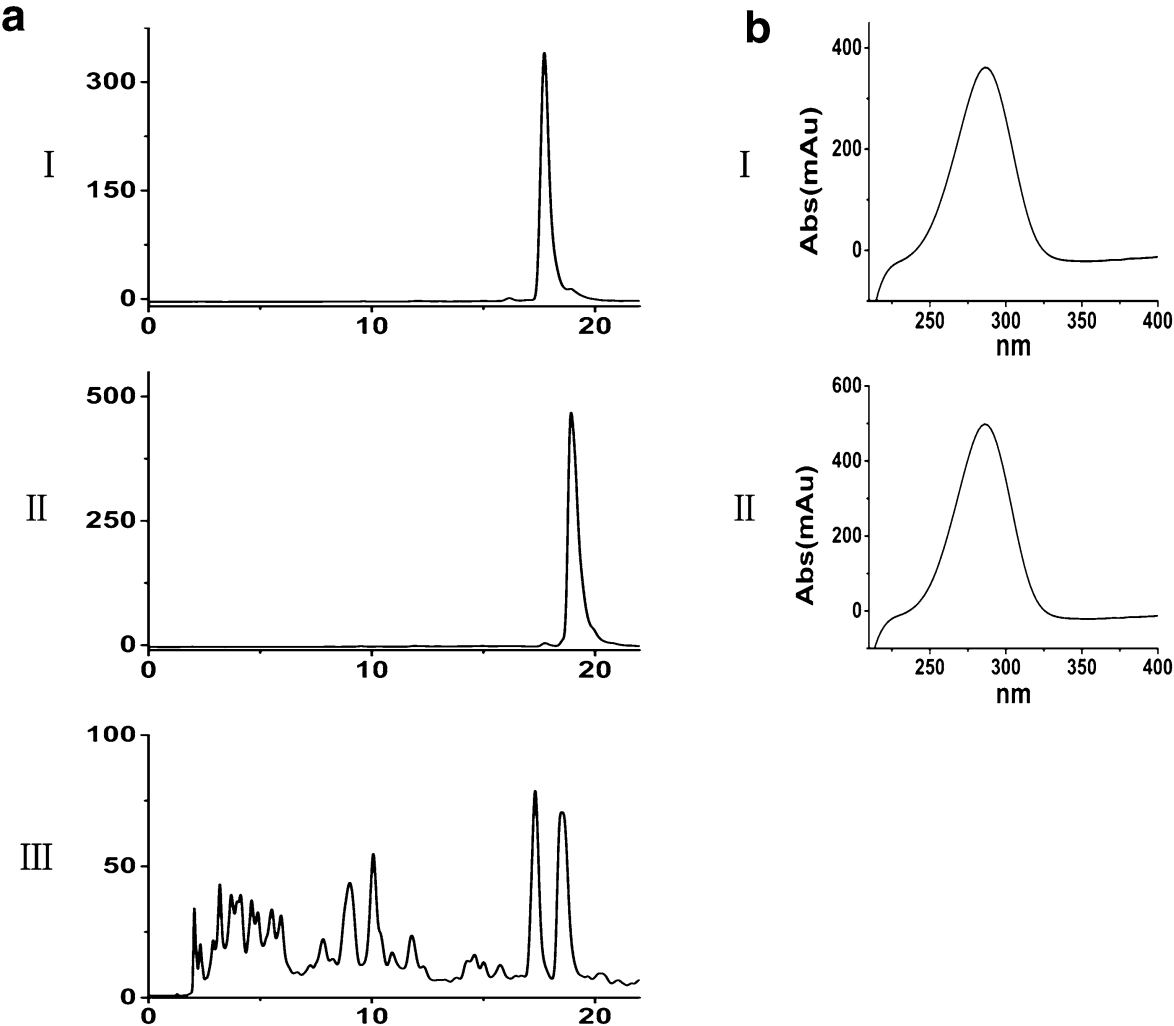
Identification of compound **1** and **2** produced by ΔwblA. HPLC chromatograms (**a**) and the UV absorption spectra of compound **1** and **2** (**b**). (*I*): purified compound **1**, (*II*): purified compound **2**, (*III*): fermentation broth from ΔwblA

Based on the fact that nikkomycin production was abolished in ΔwblA, it is noteworthy to identify whether new products could be produced by ΔwblA. The culture filtrates from the different time-course experiments were subjected to bioassays against representative gram-positive bacteria and gram-negative bacteria (Additional file 1: Table S1). The culture filtrate collected from ΔwblA after incubation for 96 h showed clear inhibition zones against both *Staphylococcus aureus* and *Bacillus cereus*, whereas no inhibition zone was found in the culture filtrate from WT (Fig. 3a, b). Chloroform extracts from these cultures were further analyzed by HPLC (Fig. 3c), and distinct peaks appeared at 17 min (compound **1**) and 18 min (compound **2**) in the extract of ΔwblA (Fig. 4a). Both compounds gave rise to distinctive absorption at wavelength 286 nm on the ultra-violet (UV) spectra (Fig. 4b), indicating that they might be new products generated by ΔwblA since these two compounds were not found in WT under the same conditions.

### Isolation and structural analyses of compound 1 and 2

To determine the chemical structures of compound **1** and **2**, 18 liters of fermentation broth of ΔwblA in SP medium was harvested and extracted with chloroform. The organic phase was concentrated and applied onto Sephadex LH-20 column for further purification. 2.3 mg of compound **1** and 5.2 mg of compound **2** were obtained after final separation by semi-preparative HPLC. The chemical structures of these two compounds were determined by Mass Spectrometry (MS) and Nuclear Magnetic Resonance (NMR) spectroscopy.

**Fig. 5:**
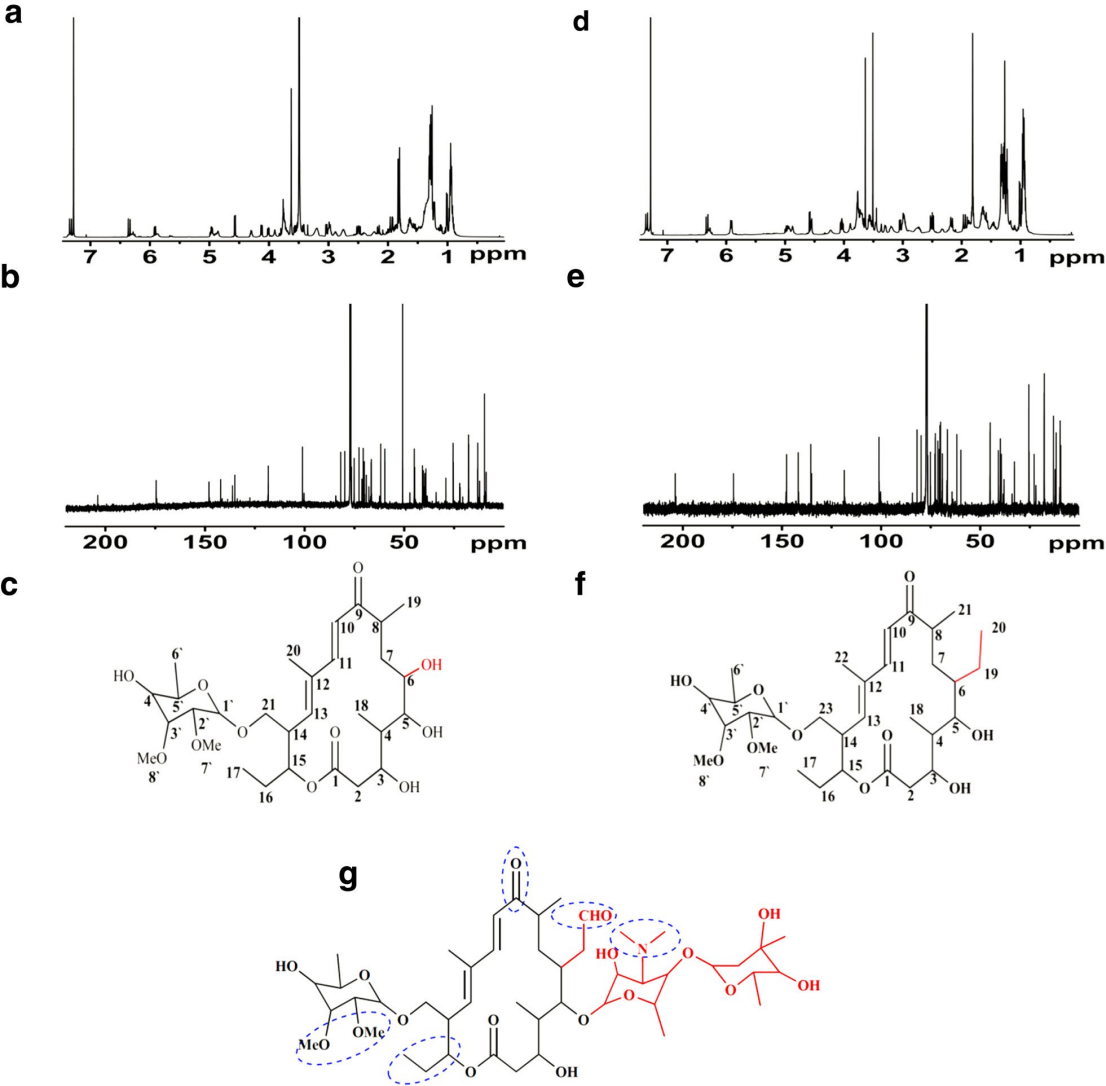
Structural determinations of compound **1** and compound **2. a** ^1^H NMR of compound **1**. **b** ^13^C NMR of compound **1**. **c** The structure of compound **1**. **d** ^1^H NMR of compound **2**. **e** ^13^C NMR of compound **2**. **f** The structure of compound **2**. **g** The structure of tylosin. Active groups contributing to tylosin activity are indicated by *dashed line*. The structural differences among tylosin, compound **1** and compound **2** are shown in* red*

High resolution positive-ion electron spray ionization mass spectrometry (HR-ESI–MS) of compound **1** gave a molecular ion peak at *m*/*z* 577.33459 ([M+Na−H_2_O]^+^) and the molecular formula was found to be C_29_H_48_O_11_. An initial survey of ^1^H NMR and ^13^C NMR spectra (Fig. 5a, b) indicated the existence of two conjugated double bonds, a mycinose moiety and two carbonyl carbons (*δ*_C_ 174.7 and 204 ppm). Cross peaks between *δ*_H_ (4.98, 1.92 and 2.5 ppm) and *δ*_C_ 174.7 ppm on HMBC indicated that compound **1** could contain a macrolide backbone. Along with other correlations (Additional file 1: Figure S1) between protons and carbons on HMBC, HSQC and ^1^H–^1^H COSY, compound **1** seemed to be an analogue of tylosin, and most signals on the lactone and mycinose moiety could be assigned based on the NMR data of tylosin, except those at positions C5 and C6 [17]. Proton and carbon resonances for the two sugar moieties at C5 and acetaldehyde group at C6 in tylosin were absent, but two additional hydroxyl groups were present as indicated by the two sets of signals (*δ*_H_ 4.1, *δ*_C_ 71.6 ppm; *δ*_H_ 4.3, *δ*_C_ 67.9 ppm), which were determined by the following analysis. A cross peak between H4 (*δ*_H_ 1.48 ppm) and *δ*_H_ 4.1 ppm on ^1^H-^1^H COSY indicated that *δ*_H_ 4.1 ppm and *δ*_C_ 71.6 ppm could be assigned to C5; while the correlation between *δ*_H_ 4.3 ppm and *δ*_C_ 71.6 ppm on HMBC suggested that *δ*_H_ 4.3 ppm and *δ*_C_ 67.9 ppm could be assigned to C6. Combined with other NMR data, compound **1** was determined as 6-hydroxy-21-O-mycinosyltylactone (Fig. 5c), a novel tylosin analogue.

For compound **2**, HR-ESI–MS gave a molecular ion peak at *m*/*z* 602.38965 ([M+NH_4_]^+^) and the molecular formula was found to be C_31_H_52_O_10_. Comparison of the ^1^H NMR and ^13^C NMR data (Fig. 5d, e) with those of compound **1** indicated a highly structural similarity between the two compounds, and the only difference is at C6. *δ*_H_ 4.3 ppm and *δ*_C_ 67.9 ppm at C6 were absent and the chemical shift at C6 was high-field shifted to *δ*_C_ 38 ppm in compound **2**. Meanwhile, two sets of extra signals (*δ*_C_ 22.7 ppm and *δ*_H_ 1.62 ppm; *δ*_C_ 9.4 ppm and *δ*_H_ 0.92 ppm) showed the existence of an ethyl group, while the correlation between *δ*_H_ 0.92 ppm and C6 (*δ*_C_ 38 ppm) confirmed that the ethyl group is attached to C6. Further analysis of the HMBC, HSQC and COSY data (Additional file 1: Figure S2), compound **2** was determined as another tylosin analogue and designated as 23-O-mycinosyltylactone (Fig. 5f).

The NMR spectroscopic data of compound **1** and compound **2** are summarized in Table 1. The structural differences among compound **1**, **2** and tylosin are illustrated (Fig. 5g).

**Table 1:** Summary of ^1^H and ^13^C NMR data for compound **1** and compound **2** in CDCl_3_

Position	Compound **1**		Compound **2**	
	*δ* (^1^H, mult., J)	^13^C (*δ*)	*δ* (^1^H, mult., J)	^13^C (*δ*)
1		174.7		174.7
2	1.92 (1H, d, 16) 2.5 (1H, dd, 17, 10.7)	39.2	1.92 (1H, d, 16) 2.5 (1H, dd, 17, 10.7)	39.2
3	3.72 (1H, d, 10.0)	67.1	3.72 (1H, d, 10.0)	66.8
4	1.48 (1H, *)	39.9	1.48 (1H, *)	39.9
5	4.1 (1H, d, 13.0)	71.6	3.77 (1H, d, 9.0)	72.6
6	4.3 (1H, *)	67.9	1.3 (1H, *)	38
7	2.0 (1H, m) 1.56 (1H, *)	29.2	1.42 (1H, m) 1.57 (1H, *)	32.7
8	2.8 (1H, br)	45.1	2.7 (1H, br)	45.1
9		204		204
10	6.34 (1H, d, 15.0)	118.3	6.33 (1H, d, 15.0)	118.6
11	7.32 (1H, d, 15.0)	148.1	7.32 (1H, d, 15.0)	147.8
12		135.1		135.5
13	5.92 (1H, d, 10.0)	141.9	5.92 (1H, d, 10.0)	141.7
14	2.98 (1H, m)	45.1	2.98 (1H, m)	45.1
15	4.98 (1H, ddd, 10.0, 10.0, 2.0)	75.3	4.98 (1H, ddd, 10.0, 10.0, 2.0)	75.2
16	1.88 (1H, m) 1.63 (1H, *)	25.4	1.88 (1H, m) 1.63 (1H,*)	25.5
17	0.94 (3H, *)	9.5	0.94 (3H, *)	9.5
18	1.0 (3H, d, 6.0)	9.5	1.0 (3H, d, 6.0)	9.6
19	1.22 (3H, d, 7.0)	17.6	1.62 (1H, *) 1.3 (1H, *)	22.7
20	1.81 (3H, s)	13.2	0.92 (3H, *)	9.4
21	4.01 (1H, dd, 9.0, 4.0) 3.55 (1H, *)	69.1	1.23 (3H, d,7.0)	17.6
22			1.81 (3H, s)	13.2
23			4.01 (1H, dd, 9.0, 4.0) 3.55 (1H, *)	69
1`	4.58 (1H, d, 7.5)	101.1	4.58 (1H, d, 7.5)	101.1
2`	3.04 (1H, dd, 7.5, 2.5)	81.5	3.04 (1H, dd, 7.5, 2.5)	81.5
3`	3.77 (1H, d, 9.0)	79.8	3.77 (1H, d, 9.0)	79.8
4`	3.2 (1H, m)	72.6	3.2 (1H, m)	72.6
5`	3.53 (1H, *)	70.5	3.53 (1H, *)	70.5
6`	1.29 (3H, *)	17.7	1.29 (3H, *)	17.7
7`	3.5 (3H, s)	59.7	3.5 (3H, s)	59.7
8`	3.63 (3H, s)	61.9	3.63 (3H, s)	61.9

In this table, *s* singlet, *d* doublet, *m* multiplet, *br* broad

* Overlapping with other signals

### Bioassays of compound 1 and 2

Structural elucidation showed compound **1** and compound **2** are 16-membered glycosylated macrolides. The functional groups responsible for the antibacterial activity of 16-membered macrolides are generally thought to be the aldehyde and the 9-keto group on the lactone, dimethylamino or methoxyl group on the sugar moieties and ethyl group at position C15 (Fig. 5g) [18]. In preliminary assays performed by disk diffusion tests, the fermentation filtrate of ΔwblA showed inhibitory activity against gram-positive pathogenic bacteria (Additional file 1: Table S1). MIC (minimum inhibitory concentration) values were then determined with purified compound **1** and compound **2** against a variety of gram-positive bacteria using tylosin as a control, a 16-membered macrolide antibiotic usually used in the treatment for various infections of animals [19, 20]. Compound **1** showed identical antimicrobial activity as compound **2**, but their activity was less than that of tylosin against most of the tested strains (Table 2). All three compounds could not inhibit the growth of *Staphylococcus epidermidis* at 100 μg/ml. However, it is intriguing that compound **1** and compound **2** significantly inhibited the growth of *S. pneumoniae* and their MICs were more than ten folds lower than that of tylosin (Table 2). The results indicated that compound **1** and compound **2** are probably promising new derivatives of tylosin for further structural optimization.

**Table 2:** Antimicrobial activities of compound **1**, **2** and tylosin

Bacteria	MIC (μg/ml)		
	Compound **1**	Compound **2**	Tylosin
*Streptococcus pneumoniae*	7.06	7.31	>100
*Streptococcus pyogenes*	3.53	3.65	0.2
*Staphylococcus epidermidis*	>100	>100	>100
*Staphylococcus aureus*	56.5	58.5	0.4
*Bacillus subtilis*	14.1	14.6	0.4
*Bacillus cereus*	28.2	29.2	0.4

## Discussion

It is imperative to find novel families of antibiotics for tackling evolving pathogens. *Streptomyces* serves as the main source of antibiotics, despite most secondary metabolic pathways are silent or poorly expressed. Based on metabolic pathways and regulatory mechanisms of antibiotic biosynthesis, specific manipulation on key gene is feasible to redirect metabolic flux to the target metabolites, such as heterologous expression of the whole cluster, repressor deletion or activator enhancement, and so on. Those approaches enabled the discovery of numerous novel antibiotics [6]. However, a large proportion of secondary metabolic pathways in *Streptomyces* have not been unveiled. Therefore, it has become necessary to devise methods and strategies to identify these valuable secondary metabolites.

WblA of *S. ansochromogenes* 7100 shares 96 % sequence identity with that of *S. coelicolor*, and is a new member of pleiotropic regulators. Disruption of *wblA* influenced the morphological differentiation and the production of antibiotics in many *Streptomyces* spp. [13, 14]. As expected, the disruption of *wblA* in *S. ansochromogenes* 7100 influenced spore formation and also abolished nikkomycin production, but led to the biosynthesis of two novel tylosin analogues. WblA can serve as a down-regulator or activator depending on the species of the strain probably via the iron-sulfur cluster in the molecule for sensing environmental signals, such as O_2_ or nitric oxide [21, 22]. In *S. ansochromogenes* 7100, WblA exerted dual function in antibiotic biosynthesis, demonstrating that the regulators of this family play important roles. Other pleiotropic regulators widely exist in many species of *Streptomyces*, such as AdpA and BldA controlling more than one pathway [23, 24]. It is applicable to obtain new compounds from the cell secondary metabolite reservoir by disrupting a single pleiotropic gene without knowing details about the mechanism or the pathway of the metabolite biosynthesis. So far, exact regulatory mechanism of WblA and its orthologues regulating antibiotics biosynthesis are still unknown.

Structure determination revealed that compound **1** and compound **2** are tylosin analogues. Tylosin can inhibit bacterial growth by binding to the large ribosomal subunit to block the peptide tunnel [25]. Despite the structure difference at C6, compound **1** and compound **2** showed similar antibacterial activity, indicating that ethyl group at C6 position is replaceable with hydroxyl group without compromising the antibacterial activity (Fig. 5g). Compared to tylosin, the activity of compound **1** and compound **2** against most indicator strains was much lower. The reduction in activity of these compounds may be resulted from the absence of some active groups contributing to the tylosin activity, such as the aldehyde at C6 position, dimethyl amino as well as the saccharide moieties at C5 (Fig. 5c, f). No inhibitory activity against *Staphylococcus epidermidis* was observed with compound **1**, compound **2** and tylosin at 100 μg/ml. However, very interestingly, compound **1** and compound **2** exhibited much higher activity against *Streptococcus pneumoniae* than tylosin (Table 2). *S. pneumoniae* strain with certain resistance to tylosin is probably due to the evolvement of pathogenic strains. Ribosome mutation is one way to obtain resistance to ribosome-targeted drugs. It was reported that replacing G2099 of ribosome with dimethyl adenine in *Haloarcula marismortui* triggered sterically clashing with dimethyl amino group linked to the saccharide moieties of tylosin and then the resistance was induced [26]. For compound **1** and compound **2**, the reduced molecular size lacking dimethyl amino and saccharide branch at C5 could be beneficial for the compound to be accommodated into the ribosome tunnel of pathogenic strains. These results suggested that compound **1** and compound **2** could serve as starting molecules for further structural optimization to produce diverse bioactive agents, which are constantly required to combat the evolving pathogens and new diseases.

## Conclusions

Two novel tylosin analogues were generated by ΔwblA. Interestingly, the activity of compound **1** and compound **2** against *S. pneumoniae* was much higher than that of tylosin. They might serve as new derivatives of tylosin for property improvement by engineering combinatorial biosynthesis of metabolic pathways.

## Methods

### Strains, plasmids, primers and growth conditions

Strains and plasmids used in this study are listed in Table 3, and the primers used in this study are listed in Table 4. *Streptomyces ansochromogenes* 7100, a natural nikkomycin producer, and its derivatives were grown at 28 °C. SP medium (3 % mannitol, 1 % soluble starch, 0.75 % yeast extract and 0.5 % soy peptone, pH 6.0) was prepared for the production of antibiotics as described previously [27]. Agar minimal medium (MM) supplemented with mannitol as sole carbon source for sporulation was prepared [28]. *Escherichia coli* JM109, routinely used as a host for propagation of plasmids, was grown in Luria–Bertani (LB) medium at 37 °C. ET12567/pUZ8002 was used for conjugal transfer of DNA from *E. coli* to *Streptomyces* [28]. Tylosin tartrate was purchased from Sigma Aldrich, and used as a control in bioassays. All fungal strains used as indicators in this study except *C. albicans* were incubated for 5 days in PDA at 28 °C. *C. albicans* was grown in PDA for overnight at 37 °C.

**Table 3:** Strains and plasmids used in this study

Name	Description	Sources
Strains		
*S. ansochromogenes* 7100	Wild-type strain	[27]
ΔwblA	The ORF of *whlA* consists of 339 bp, and 230 bp of them was replaced by kanamycin resistance gene (*neo*)	This study
ΔwblA/pSET152::*wblA*	The complemented strain of ΔwblA	This study
*Escherichia coli* JM109	*recA1*, *endA1*, *gyrA96*, *thi*-*1*, *hsdR17*, *supE44*, *relA1*, Δ(lac-proAB)/F’ [traD36, proAB + lacIq, lacZΔM15]	Invitrogen
*Escherichia coli* ET12567/pUZ8002	*dam dcm hsdS cat tet*/pUZ8002	[31]
*Staphylococcus aureus* CGMCC1.89	Indicator strain for bioassays	CGMCC
*Bacillus subtilis* CGMCC1.1630	Indicator strain for bioassays	CGMCC
*Bacillus cereus* CGMCC1.1626	Indicator strain for bioassays	CGMCC
*Candida albicans* CGMCC2.4159	Indicator strain for bioassays	CGMCC
*Alternaria longipes* CGMCC3.2946	Indicator strain for bioassays	CGMCC
Plasmids		
pwblA-DM	Plasmid used for the construction of ΔwblA	This study
pSET152:: *wblA*	pSET152 containing the intact *wblA* with its putative promoter	This study
pSET152	Integrative vector	[32]
pKC1139	*E. coli*-*Streptomyces* shuttle vector	[28]
pBluescript KS+	Routine cloning and subcloning vector	Stratagene

*CGMCC* China General Microbiological Culture Collection Center

**Table 4:** Primers used in this study

Primers	Sequence (5′-3′)
Primers for gene disruption and complementation	
LwblA-F	AAGCTTTCGGGTACGCCATCTCGTA
LwblA-R	TCTAGAGCTGCTCCCTGAACGAACA
RwblA-F	GGATCCACGACGAGGTGTACGAGAAC
RwblA-R	GATATCTGACGCTGCTGGAGGAGAT
Kan-F	TCTAGAGATCCCCTGGATACCGCTCG
Kan-R	GGATCCGTACCCGAACCCCAGAGTC
wblAJ-F	AACTGGCGGCGGTGAATA
wblAJ-R	ACGGACGGAGCACATATAGG
CwblA-F	GGATCCGCCTGAACGGACGGAGCACATA
CwblA-R	TCTAGAAGCACACTGACACCGAGGAACTTGGC
Primers for qRT-PCR	
RTsanG-F	GGCGTACACAGCTCAAGAGC
RTsanG-R	AATTCGTCGATGAGCTGATC
RTsanN-F	AGATCATGCGCTCGGACTGT
RTsanN-R	TGGCGTGCAGGATCGGTA
RTsanO-F	ACTGCGATCCGTGGTCAA
RTsanO-R	TGTACTCCAGGCACTCCC
RTsanF-F	CGGCGCTGGAGGAACGTAC
RTsanF-R	GGGTGTAGAGGCCGATGCT
RThrdB-F	GCTGGCCAAGGAACTCGACAT
RThrdB-R	CGAAGCGCATGGAGACGACG

### Construction of recombinant strains

To construct the *wblA* disruption mutant (ΔwblA) of *S. ansochromogenes* 7100, the DNA fragment corresponding to the upstream region of *wblA* was amplified by PCR using primers LwblA-F and LwblA-R, and then it was digested with *Hin*dIII and *Xba*I. The pwblA1 was constructed by inserting above PCR product into the same sites of pKC1139. The DNA fragment corresponding to the downstream region of *wblA* was amplified by PCR using primers RwblA-F and RwblA-R, followed by digestion with *Bam*HI and *Eco*RV and inserted into the same sites of pwblA1 to generate pwblA2. Kanamycin resistance gene was amplified by PCR using primers Kan-F and Kan-R followed by digestion with *Bam*HI and *Xba*I, and inserted into the same sites of pwblA2 to generate pwblA3. Subsequently, pwblA3 was introduced into *S. ansochromogenes* 7100 via ET12567/pUZ8002 by conjugal transfer. The transformants resistant to kanamycin (Kan^r^) but sensitive to apramycin (Apr^s^) were selected and further confirmed by PCR using primers wblAJ-F and wblAJ-R. For complementation analysis, the fragment containing the intact *wblA* with its putative promoter region was amplified using primers CwblA-F and CwblA-R, and inserted into the *Eco*RV site of pSET152 to generate pSET152::*wblA*. Subsequently, pSET152::*wblA* was introduced into ΔwblA by conjugal transfer, and the resulting complemented strain was further confirmed by PCR. The null mutant was constructed by integrating pSET152 vector into the chromosome of ΔwblA as a control. All PCR amplicons were confirmed by sequencing.

### RNA isolation and qRT-PCR

Total RNA was isolated from *Streptomyces*, and quantitative Real Time PCR (qRT-PCR) was performed as described previously [29].

### Microscopy

For scanning electron microscopy, colonies were fixed in 2.5 % (v/v) glutaraldehyde for 4 h, stained with osmic acid for 2–4 h and dehydrated with ethanol at different concentrations. Each sample was coated with platinum-gold and then examined with a Hitachi D-570 scanning microscope.

### Detection of nikkomycin and tylosin analogues

Nikkomycin was detected by disk agar diffusion and HPLC as previously described [30]. The detection of tylosin analogues was performed by HPLC on an Agilent 1260 system equipped with a ZORBAX SB-C18 reverse phase column (4.6 × 250 mm, 5 μm, Agilent). Samples were eluted at 1 ml/min with a linear gradient from 50 to 80 % of methanol in water over 25 min at wavelength of 280 nm. Each experiment was performed in triplicate.

### Isolation and structural determination of compound 1 and 2

For antibiotics production, spore suspensions were inoculated into liquid SP medium and cultured at 28 °C for 24 h as seed culture in shake flask (220 revolutions per minute, rpm), and then 30 ml of seed culture was transferred to 3 L of SP in a 5 L fermentor (BIOTECH-5JG, BX-BIO). BIOTECH-FCS software was used to control the equipment and collect data. Air was sparged into the fermentor to supply oxygen at four times atmospheric pressure, and the rotor speed was 400 rpm. After fermentation for 5 days at 28 °C the culture broth of ΔwblA was filtered by Pyrex Buchner funnel with a fritted disc (pore size 40–60 mm). Then the supernatant was extracted by separatory funnel with equal volume of chloroform for three times at room temperature. Chloroform extract was evaporated to dry. The resulting sample was re-dissolved in methanol and then separated on Sephadex LH-20 as mentioned above. Active fractions were collected and purified by semi-preparative HPLC equipped with ZORBAX SB-C18 reverse phase column (9.4 × 250 mm, 5 μm, Agilent) by linear gradient elution as mentioned above.

MS analysis was performed on LTQ Orbitrap hybrid mass spectrometer (Thermo-Fisher) equipped with a Dionex Ultimate 3000 nano-flow system and a nano-electrospray ion source. NMR spectra were recorded on a 500 MH_Z_ Bruker spectrometer using CDCl_3_ as the solvent.

### Determination of minimum inhibitory concentration (MIC)

Compound **1**, **2** and tylosin standard were dissolved in DMSO and serially diluted with LB prior to mixing with indicator strains. Indicator strains were pre-incubated in LB on a rotary shaker at 37 °C for overnight. Assays for determining MIC were performed on 96-well plates consisting of the diluted compounds, indicator strains (0.5 %) and 0.5 % DMSO. Strains growing in LB medium containing 0.5 % DMSO without test compounds were used as positive controls, and LB medium containing 0.5 % DMSO was used as negative control. The growth of indicator strains was measured after 12 h of incubation for *S. epidermidis, S. aureus, B. subtilis* and *B. cereus*, and 24 h for *S. pneumoniae* and *S.pyogenes* on a microplate reader (Synergy H4, Biotech) at wavelength of 600 nm. Each experiment was performed in triplicate.

## Additional file

10.1186/s12934-015-0359-5 **Figure S1.** NMR Spectra of compound **1**. (A) Summary of key correlations between protons and carbons in compound **1** based on NMR spectroscopic data. (B) ^1^H-^1^H COSY spectrum of compound **1**. (C) ^1^H-^13^C HSQC spectrum of compound **1**. (D) ^1^H-^13^C HMBC spectrum of compound **1**. **Figure S2.** NMR Spectra of compound **2**. (A) Summary of key correlations between protons and carbons in compound **2** based on NMR spectroscopic data. (B) ^1^H-^1^H COSY spectrum of compound **2**. (C) ^1^H-^13^C HSQC spectrum of compound **2**. (D) ^1^H-^13^C HMBC spectrum of compound **2**. **Table S1.** Antimicrobial activities of fermentation broth from *S. ansochromogenes* 7100 and ΔwblA by agar diffusion assays.
