# Supplementary material for: Identification of novel tylosin analogues generated by a wblA disruption mutant of Streptomyces ansochromogenes
Source: Microb Cell Fact. 2015 Nov 2;14:173. doi: 10.1186/s12934-015-0359-5 (PMC4630966; doi:10.1186/s12934-015-0359-5)
Supplement: Supplementary file 1 — 10.1186/s12934-015-0359-5 Figure S1. NMR Spectra of compound 1. (A) Summary of key correlations between protons and carbons in compound 1 based on NMR spectroscopic data. (B) 1H-1H COSY spectrum of compound 1. (C) 1H-13C HSQC spectrum of compound 1. (D) 1H-13C HMBC spectrum of compound 1. Figure S2. NMR Spectra of compound 2. (A) Summary of key correlations between protons and carbons in compound 2 based on NMR spectroscopic data. (B) 1H-1H COSY spectrum of compound 2. (C) 1H-13C HSQC spectrum of compound 2. (D) 1H-13C HMBC spectrum of compound 2. Table S1. Antimicrobial activities of fermentation broth from S. ansochromogenes 7100 and ΔwblA by agar diffusion assays. [file 12934_2015_359_MOESM1_ESM.docx]

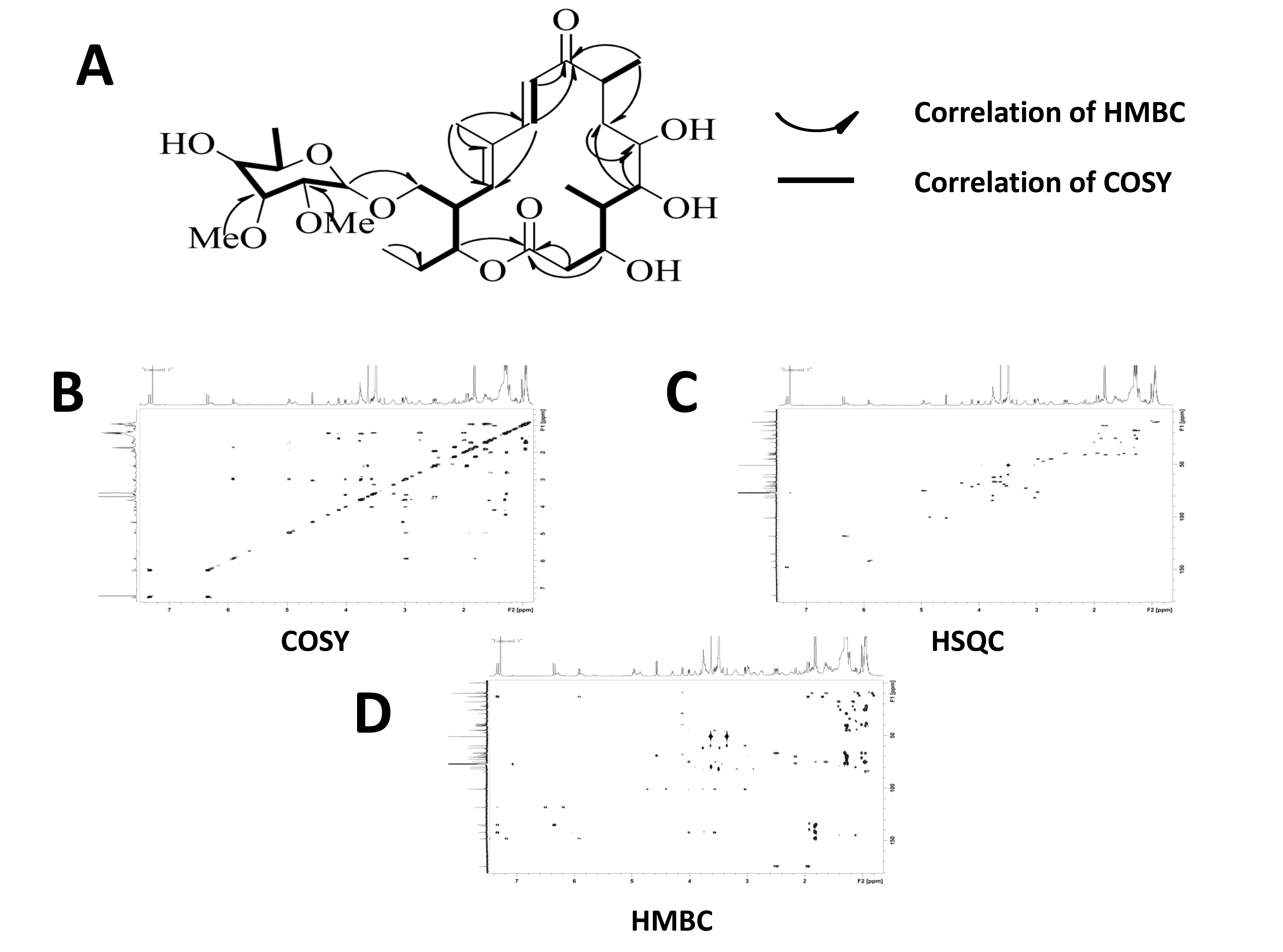


**Figure S1 NMR Spectra of compound 1.** **(A)** Summary of key correlations between protons and carbons in compound **1** based on NMR spectroscopic data. **(B)** ^1^H-^1^H COSY spectrum of compound **1**. **(C)** ^1^H-^13^C HSQC spectrum of compound 1. **(D)** ^1^H-^13^C HMBC spectrum of compound **1**.


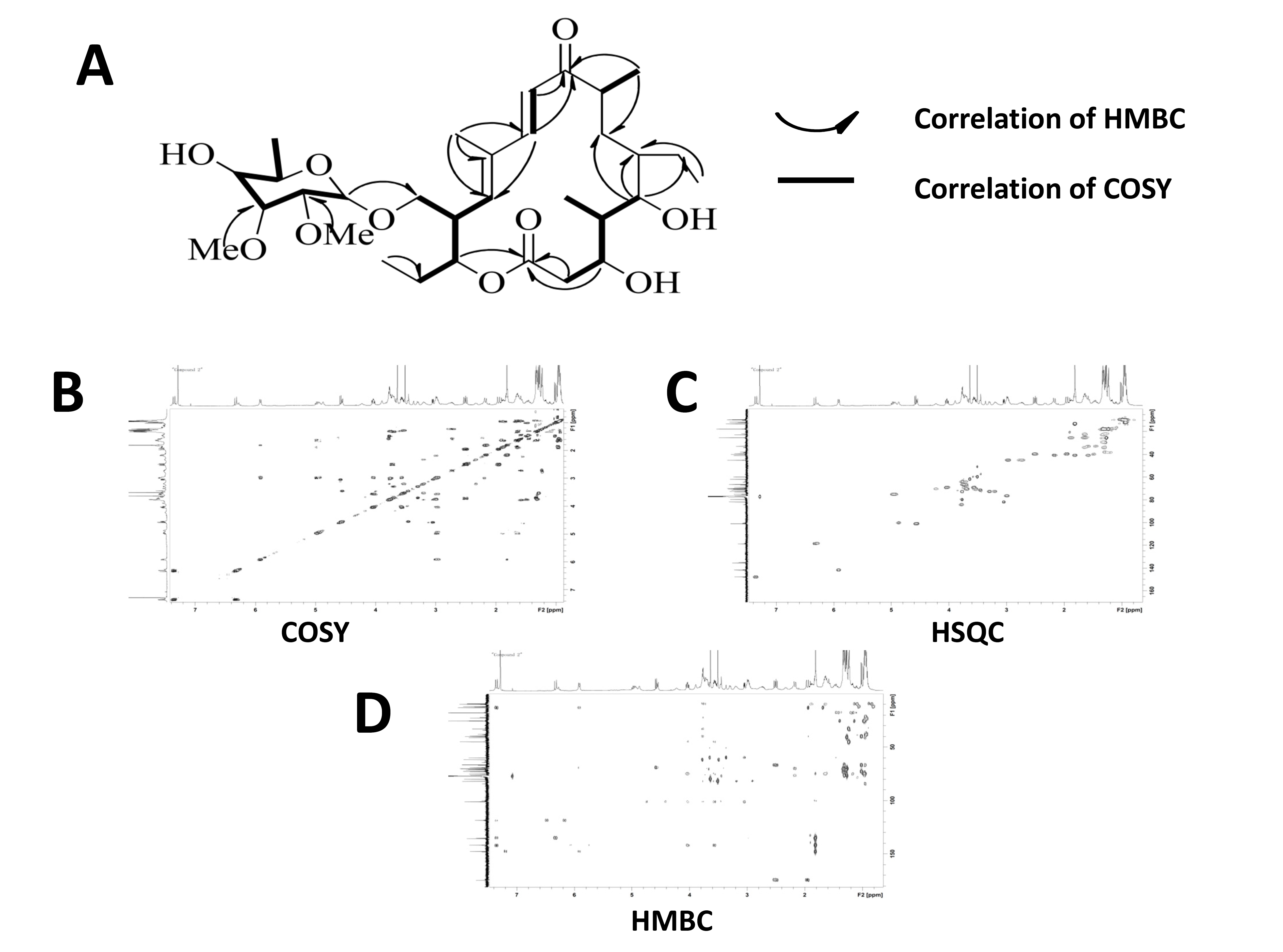


**Figure S2 NMR Spectra of compound 2.** **(A)** Summary of key correlations between protons and carbons in compound **2** based on NMR spectroscopic data. **(B)** ^1^H-^1^H COSY spectrum of compound **2**. **(C)** ^1^H-^13^C HSQC spectrum of compound **2**. **(D)** ^1^H-^13^C HMBC spectrum of compound **2**.

**Table S1 Antimicrobial activities of fermentation broth from *S. ansochromogenes* 7100 and ΔwblA by agar diffusion assays**

| **Strains** | **WT** | ΔwblA |
| --- | --- | --- |
| *Staphylococcus aureus* | **-** | **+** |
| *Bacillus subtilis* | **-** | **+** |
| *Bacillus cereus* | **-** | **+** |
| *Escherichia coli* | **-** | **-** |
| *Pseudomonas aeruginosa* | **-** | **-** |
| *Candida albicans* | **+** | **-** |
| *Magnaporthe grisea* | **+** | **-** |
| *Alternaria longipes* | **+** | **-** |
| *Cryphonectria parasitica* | **+** | **-** |
| *Sporisorium scitamineum* | **-** | **-** |
